# Effects of Xiaoyaosan on Depressive-Like Behaviors in Rats With Chronic Unpredictable Mild Stress Through HPA Axis Induced Astrocytic Activities

**DOI:** 10.3389/fpsyt.2020.545823

**Published:** 2020-10-20

**Authors:** Ming Song, Jianjun Zhang, Xiaojuan Li, Yueyun Liu, Tingye Wang, Zhiyi Yan, Jiaxu Chen

**Affiliations:** ^1^ School of Traditional Chinese Medicine, Beijing University of Chinese Medicine, Beijing, China; ^2^ Formula-pattern Research Center, School of Traditional Chinese Medicine, Jinan University, Guangzhou, China

**Keywords:** Xiaoyaosan, depression, astrocyte, glial fibrillary acidic protein, chronic unpredictable mild stress, hippocampus

## Abstract

**Abstract:**

Astrocytes in the hippocampus are immediately relevant to depressive-like behavior. By regulating their activities, Xiaoyaosan (XYS), a traditional Chinese medicine compound, works in the treatment of depression.

**Objective:**

Chronic unpredictable mild stress (CUMS) rat model was established to observe the regulation of XYS. We investigated the behavioral changes of CUMS, the expression of corticosterone (CORT) of the hypothalamo–pituitary–adrenal (HPA) axis, the expression of Glu-NMDA receptor and astrocytes glial fibrillary acidic protein (GFAP) in the hippocampus. We also investigated whether these changes were linked to XYS.

**Methods:**

80 adult SD rats were randomly divided into four groups, control group, CUMS group, XYS group, and fluoxetine group. The rats in the control group and the CUMS group received 0.5 ml of deionized water once a day by intragastrically administration. Rats in the two treatment groups received XYS (2.224g/kg/d) and fluoxetine (2.0mg/kg/d) once a day, respectively. Rat hippocampus GFAP and Glu-NMDA receptor were respectively detected by real-time fluorescent quantitative PCR and western blot. The CORT of HPA axis was detected by Elisa. Body weight, food intake, and behavioral tests, such as open field tests, the sucrose preference test, and exhaustive swimming test, were used to assess depressive-like behavior in rats.

**Results:**

In this work, significant behavioral changes and differences in expression of the CORT of HPA axis and hippocampal GFAP and Glu-NMDA receptor were presented in CUMS-exposed rats. Like fluoxetine, XYS improved CUMS-induced rat’s body weight, food intake, and depressive-like behavior. The study also proved that XYS could reverse the CUMS-induced changes of the CORT of HPA axis and affect the astrocytic activities and down-regulate the NR2B subunit of NMDA receptor (NR2B) level in the hippocampus.

**Conclusion:**

Changes in the hippocampus GFAP and Glu-NMDA receptor may be an essential mechanism of depression. Besides, XYS may be critical to the treatment of depression by intervention the HPA axis, GFAP and Glu-NMDA receptor.

## Introduction

Due to the accelerated pace of life, depression is a common mental health problem in modern society (1). Under chronic stress, the neuroendocrine system of the body will produce pathophysiological changes (2), such as pyramidal cell atrophy, astrocyte dysfunction, and related signaling pathway changes that cause the hippocampus nerve regeneration disorders. It also causes memory loss, anxiety, and behavioral abnormalities, and also causes depressive symptoms or aggravating the emotional chaos of depressed patients (3, 4).

The causes of depression are very complicated, including social and psychological factors that affect the progress of the disease. Chronic stress is one of the highest risk factors for depression (5). Astrocytes are the most abundant and widely distributed cells in the central nervous system (6, 7). In recent years, we found that astrocyte plays an increasingly important role in the occurrence and development of neuropsychiatric diseases (8). At present, research on astrocyte has become a hot spot in the study of the pathophysiological mechanism of depression and even other neuropsychiatric disorders, such as epilepsy and Alzheimer’s disease. The regulation of astrocytes is expected to become an essential target for the prevention and treatment of depression (9).

Besides, some studies have found that the number of astrocytes in the brain and the expression of its specific cytoskeleton protein Glial Fibrillary Acidic Protein (GFAP) are significantly decreased in some young patients with early-onset depression, and the expression level of GFAP can increase with age (10). GFAP is a biological marker protein of astrocytes (11). Numerous studies have shown that GFAP is involved in various physiological functions of astrocyte, such as maintaining the blood-brain barrier, synaptic plasticity, cell proliferation, and regulating the transport of vesicles and lysosomes in astrocyte (12). Decreased levels of GFAP protein in key brain regions such as the cortical, limbic system and hippocampus in patients with depression may be the critical cause of astrocyte and neuronal apoptosis, as well as specific brain changes in the brain. Furthermore, GFAP can enter the blood circulation from the brain through the blood-brain barrier, so the changes in protein levels of GFAP in the central nervous system and peripheral blood can reflect the degree of astrocyte damage in neurodegenerative diseases. At present, GFAP has become a new target for the occurrence, development, and treatment of depression (13, 14). But whether it affects the astrocyte and HPA axis is still unknown.

Xiaoyaosan (XYS) is a well-known traditional Chinese medicine formula composed of Bupleurum 30 g, Angelica 30 g, Radix Paeoniae Alba 30 g, Atractylodes 30 g, Poria 15 g, Zhigancao 15 g, Rhizoma Zingiberis Recens 10g, Mint 10 g. XYS was first described in the “Taiping Huimin Heji Jufang” and has been used to treat various diseases for hundreds of years and also extensively used in clinical and experimental research of depression (15). Previous studies have shown that XYS exerts an antidepressant-like effect by regulating brain regions such as the hippocampus, hypothalamus and locus coeruleus. My laboratory has been engaged in animal research on depression (3). We have established a CUMS depressive rat model and found that the classic compound XYS has a significant regulatory effect. It can works in a variety of neural circuits, with multiple targets, a multi-directed role (16). However, whether the GFAP and Glu-NMDA receptor is regulated, and the current mechanism is still unclear.

Some scholars have suggested that the Glu-NMDA receptor-NO pathway in the hippocampus may be damaged when the brain is stressed (17). The hippocampus limbic system has a clear role in declarative memory and spatial learning (18). Hippocampus is associated with the inhibition of the HPA axis and is susceptible to chronic stress, aging, stroke, and brain trauma (19). The hippocampus atrophy in patients with depression is associated with symptoms such as cognition and emotion (20). Chronic stress increases extracellular glutamate (Glu) concentration in the hippocampus may be a significant cause of neuronal atrophy and death (21). Glutamate is the most critical type of excitatory neurotransmitter in the brain. It is vital for learning, memory, and emotion (22). When the body is overwhelmed by stress, the HPA axis activates, resulting in the increase in glutamate release and secretion, which increases the excitability of the brain, increases the body’s ability to adapt to stress and stimulation. It also leads to an increase in glucocorticoid, and strength of the body to overcome the stress. Increased sensitivity, this behavior is called “unsteady load”, but it also causes neurotoxicity in the hippocampus. As the stimulation continues, it eventually causes necrosis of hippocampus neurons (23, 24).

Therefore, this paper puts forward the hypothesis: Chronic stress leads to hyperfunction of the HPA axis, followed by increased CORT content to a certain degree. Glu system is moderately activated, and the body’s adaptability is enhanced. However, with the over-activation of the Glu system, the astrocyte (GFAP) content of hippocampal is reduced and neuron injury, eventually leading to the occurrence of depression. Furthermore, this study focused on the relationship between hippocampus neurons and astrocytes, and further observed the regulatory role of traditional Chinese medicine XYS.

## Materials and Methods

### Animals

80 healthy male Sprague Dawley (SD) rats with the body of 180–200 g (12-week old; SCXK(JING)2016-0006 were acquired from Beijing Vital River Laboratory Animal Technology Co., Ltd. (Beijing, China) and then fed in a standard animal room (room temperature: 20°C–24°C; relative humidity: 30%–40% and light condition: 12-h dark/light cycle).

The experiment was approved by the Institutional Animal Care and Use Committee at Beijing University of Chinese Medicine and complied with the Animal Management Rules of the Chinese Ministry of Health and existing current animal welfare guidelines (NO. BUCM-4-2013101501-4001).

### Preparation of Drugs

According to a previous publication (15), the traditional Chinese medicine compound stems from “Taiping Huimin Heji Jufang” in XYS (Bupleurum 30 g, Angelica 30 g, Radix Paeoniae Alba 30 g, Atractylodes 30 g, Poria 15 g, Zhigancao 15 g, Rhizoma Zingiberis Recens 10 g, Mint 10 g). All of the raw herbs were provided by Jiuzhitang Co., Ltd. and processed the herbs into a dry extract in Beijing University of Traditional Chinese Medicine under the Regulation on Processing of Traditional Chinese Medical Herbal Pieces of Beijing. Meanwhile, we have previously used an HPLC-LTQ-Orbitrap-MS eluted system to identify eight compounds from XYS samples (25), which matched the corresponding peaks in XYS. Fluoxetine hydrochloride capsule was employed in the experiment, Patheon France, packaged by Lilly Suzhou Pharmaceutical Co., Ltd. 20mg/granule. Product Lot Number 5545A.

### CUMS Procedure and Drug Administration

#### Experimental Grouping

After seven days of adaptive feeding, the weight was labeled, and then 80 rats were randomly divided into four groups by Excel. There were 20 rats in each group: control group, model group, XYS group, and fluoxetine group.

#### Modeling

**Figure 1:**
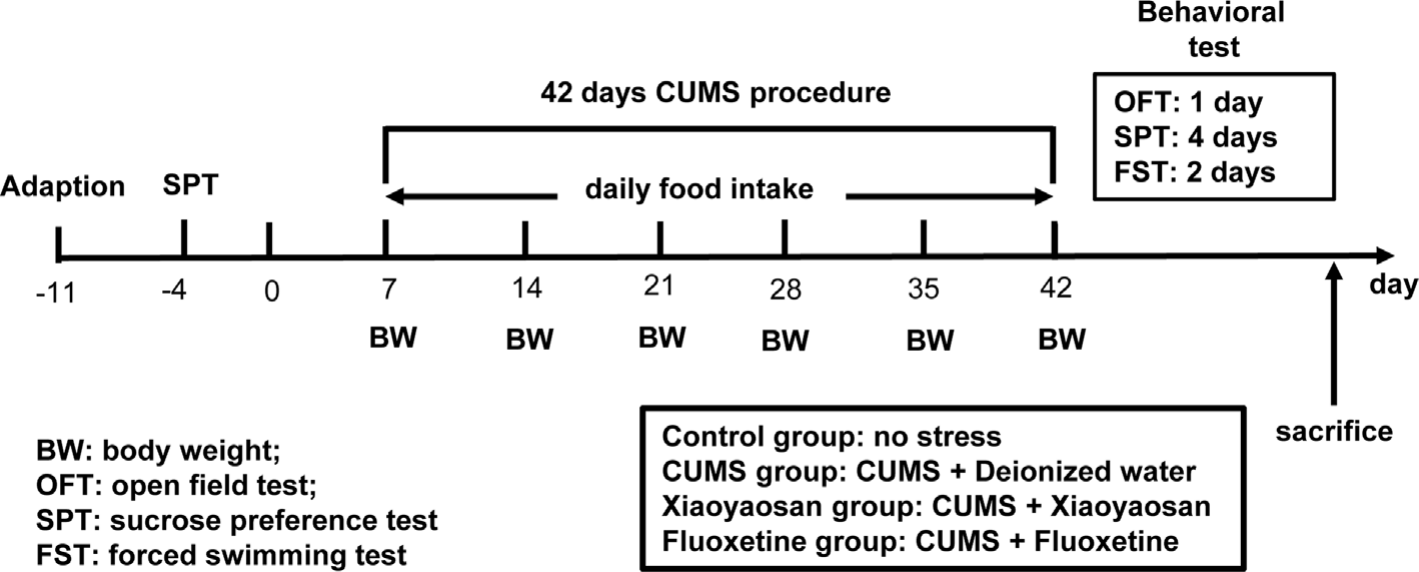
The entire experimental plan. First, one week of adaptive feeding, sucrose preference test (SPT) was conducted as a baseline to determine the initial behavioral condition of the rats. During the 42 days chronic unpredictable mild stress (CUMS) procedure, rats in each group received the respective treatment. Open field test (OFT), SPT, and forced swimming test (FS)T were carried out to detect depressive-like behavior in rats after CUMS modeling. After completing all behavioral tests, animals were sacrificed, and tissue was collected.

The model of rats with depression was replicated by CUMS. Selected items include (1) 45°C baking 5 min (Place the rat single cage under a 45°C electric heating lamp for 5 min), (2) 4°C swim 5 min (4°C ice water swimming for 5 min), (3) 85 dB noise 5h (85 db white noise stimulation for 5 h), (4) bondage stress 3h (Restrictive restraint for 3 h), (5) strange smell 24 h (Spray glacial acetic acid on the litter for 24 h), (6) strange objects 17 h (Put some plastic toys in the rat cage for 17 h) and (7) damp bedding 17 h (Put in damp litter for 17 h). Each model is selected every day, and each stress item is not displayed continuously. The CUMS random stress timetable is shown in **Table 1**. The experimental schedule is presented in **Figure 1**.

**Table 1:** CUMS random stress timetable.

Date	45°C baking	4°C swimming	85 dB noise	Bondage stress	Strange smell	Strange objects	Damp bedding
Monday	√						
Tuesday		√					
Wednesday			√				
Thursday				√			
Friday					√		
Saturday						√	
Sunday							√

#### Administration

The rats in the control group and the CUMS group received 0.5 ml of deionized water once a day by intragastrically administration. The rats in the two treatment groups received XYS (2.224 g/kg/d) and fluoxetine (2.0 mg/kg/d), respectively. From the first day of modeling, the rats in each group were intragastrically administered at 30-60 min after modeling and converted into equivalent drug doses of rats according to the adult body weight of 60 kg, the treatment of rats was 0.161/60 kg for adults and 2.224 g/kg/d for crude drugs. The fluoxetine group was administered with deionized water in an amount of 0.2 mg/100g (2.0 mg/kg/d) of body weight. The XYS powder was dissolved in deionized water. The CUMS group and control group were given the same amount of deionized water and were intragastrically administered at the end of the modeling. The deionized water or drugs were intragastrically administered for 42 days.

#### Sucrose Preference Test (SPT)

The SPT was performed at two steps, day 0 and day 42. Two days before the experiment, the rats were trained to adjust to drinking the sugar water. In the first step, the rats of each group were raised in single cages. Two bottles of 1% sucrose solution (sugar water) were placed in each cage, and the rats were allowed to drink freely for 24 h. Then, place a bottle of 1% sucrose solution and a bottle of purified water in each cage, and allow the rats to drink freely for 24 h. This period was the training period. The second step was to place 1 bottle of 1% sucrose solution and 1 bottle of purified water in each cage, allowing rats to drink freely for 1 h, and record the weight of the sucrose solution and purified water before and after the experiment. Sugar water consumption rate=sugar water consumption/total liquid consumption×100%. This experiment was used primarily to evaluate the degree of loss of pleasure in rats.

#### Forced Swimming Test (FST)

The forced swimming experiment was carried out in two steps: the top of a cylindrical transparent glass bucket (50 cm high and 20 cm diameter) was opened, and purified water (35 cm, 20–25°C) was added to the bucket. Mice were placed gently into the water and swimming behavior is recorded for 15 min, as a training period before the formal experiment, the formal experiment is conducted 24 h after the training is completed. The operator recorded the immobility time of each group of rats in the bucket within 5 min to evaluate the depressive behavior of each group of rats. The FST is the most extensive trial used to evaluate the effect of antidepressants.

#### Open Field Test (OFT)

Rats were subjected to OFT to evaluate the behavioral characteristics of spontaneous activity, exploration, anxiety. The OFT was performed respectively at day 0 and day 42. The rats were measured in a 40cm×40cm×15cm wooden box without ceiling, the floor was divided into 5×5 squares by white lines, each rat was placed right in the center and record the movement condition for 5 min with HD camera. Before the formal experiment, the rats in each group were moved to the operation room 30 min in advance. The operator gently placed the rats into the medium box of the field box and recorded the number of standing rats in each group within 5 min. At the same time, Etho Vision 3.0 software was used to analyze the behavioral parameters of the rats in each group, such as the total moving distance. At the end of each experiment, the cellar box was cleaned with 75% ethanol and water. After the odor was dispersed, the next test was performed, and the whole experiment was kept quiet. The total distance moved within 5 min, the number of crossings which at least three feet entering a square at the same time are counted as one time, and the number of groom times within 5 min were calculated.

### Sample Collection and Preparation

After modeling and behavioral test, serum and the hippocampus tissues from five rats in each group were collected for protein analysis, and then collected the other five rats in each group with RNA preservation solution (Biotech, #2714) for Quantitative Real-Time Polymerase Chain Reaction (RT-qPCR) assay.

### Enzyme-Linked Immunosorbent Assay (Elisa) Analysis

The serum CORT levels were determined with a commercial Elisa kit (CORT, Enzo ADI-900-097, New York, NY, USA). First of all, add 100 μl of each standard or sample to a 96-well plate depending on the manufacturer’s instructions. Wrap with primary antibody and incubate at 37°C lasts for 1 h. Then wash holes with water 0.05% Tween 20 (PBST). After incubation and washing several times, 100 μl of 1:1000 horseradish peroxidase (HRP)-conjugated secondary antibody diluted in PBST was added to each well, and the plates were incubated at 37°C. for 1 h. Then, all wells were washed three times with PBST and produced with 100 μl of TMB (3,3’,5,5’-tetramethylbenzidine solution) substrate per well until yellow appears. The reaction was stopped by the addition of 2 mol/L H_2_SO_4_. Read a sample absorbance at 450 nm using a Multiskan^TM^ GO (Thermo Fisher Scientific, Waltham, MA, USA) detector system. Hippocampus Glu was determined by using a commercial kit (Glu, SuoQiao, shanghai, SH, CH). About 0.2 g of hippocampus tissue was weighed, 2 ml of reagent one was added, and homogenization was carried out in an ice bath. Centrifuge at room temperature for 10 min, take the supernatant and place it on ice for testing. Then, the spectrophotometer was preheated for more than 30 min, the wavelength was adjusted to 570 nm, and the distilled water was zeroed. Add the following reagents to the covered EP tube:

**Table:** 

The name of the reagent (μl)	Measuring tube	Control tube
Sample	1,000	
Reagent I		1,000
Reagent II	200	200

Mix well, 90°C water baths for 20 min (tighten to prevent water dispersion loss), water cooling, colorimetric at 570nm wavelength, △A=A determination tube - A control tube. The regression equation was determined under standard conditions at y= 0.0074x - 0.5255; x is the glutamine acid content (μg/ml), and y is the absorbance.

### RT-qPCR Analysis

Next, we continue to explore changes in GFAP and NR2B mRNAs in the hippocampus. The total RNA in the hippocampus of each rat was extracted using Trizol reagent (Thermo Fisher Scientific, Waltham, MA, USA). The concentration of total RNA was determined by a spectrophotometer (Eppendorf, Germany), and the purity of RNA was measured by 1% agarose gel electrophoresis. The RNA from each sample was utilized to synthesize the first-strand cDNA using a Revert Aid First Strand cDNA Synthesis Kit (Thermo Fisher Scientific, Waltham, MA, USA) on a C1000 TouchTM Thermal Cycler (Bio-Rad, California, CA, USA). The sequences for primers showed in **Table 2** were designed based on published mRNA sequences in NCBI, and then synthesized by a specialized biotechnology company (Sangon Biotech Co., Ltd., Shanghai, China). The SYBR^®^ Green PCR Master Mix (Thermo Fisher Scientific) was used to amplify the cDNA in the Multicolor Real-time PCR Detection System (Bio-Rad Laboratories Inc.), and the cycling parameters were as follows: 95°C for 10 min, then 40 cycles of 95°C for 15 s and 55°C (GFAP) or 58°C (NR2B) for 1 min, respectively, then followed by 65°C for 5 s and 95°C for 15 s. The 2^−ΔΔCt^ method was used to calculate the results.

**Table 2:** Primer sequences used in the RT-qPCR analysis.

Gene	Sequences
**GFAP** Forward Reverse **NR2B** Forward Reverse	5′-GACCTGCGACCTTGAGTCCT-3′ 5′-AGCGAGTGCCTCCTGGTAAC-3′ 5′-AGCTTCCGTCATGCTCAACA-3′ 5′-CGATGGTACTGCGGATCTTG-3′

### Western Blot (WB) Analysis

Protein levels of the GFAP and NR2B in the hippocampus were identified by WB. Hippocampal tissues were used to prepare the total proteins with RIPA Lysis buffer (Biomiga, Santiago, CA, USA). According to the molecular weight of the target protein GFAP (50 kDa) and NR2B (166 kDa), 12% and 5% SDS-PAGE gels were selected, and then proteins were transferred onto polypropylene fluoride (PVDF) membranes. The 5% nonfat milk was used to block the membranes and dilute antibody, and the primary antibodies were anti-GFAP antibody (60190-1-lg, Mouse polyclonal to GFAP, diluted 1:5,000; Proteintech Group, Rosemont, IL, USA), anti-GRIN2B antibody (21920-1-AP, rabbit polyclonal to NR2B, diluted 1:500; Proteintech Group, Rosemont, IL, USA) and β-actin monoclonal antibody (66009-1lg, mouse monoclonal to β-actin, diluted 1:5,000; Proteintech Group, Rosemont, IL, USA). The enhanced chemiluminescence (ECL) detection reagent and Thermo Fisher Scientific was used to develop the membranes for 2 min, and then the Tanon-5200 system (Tanon, Shanghai, China) was used for exposure. The intensity of the protein band was read by Tanon Gis software (Tanon).

### Statistical Analysis

All dates were expressed as means ± standard errors of the means (SEM). And analyzed the date by SPSS 21.0, one-way analysis of variance (ANOVA), or non-parametric test was used for data processing based on normality test and homogeneity test for variance and least significant difference (LSD) method was adopted for the comparisons between groups. Furthermore, a multivariate analysis process of variance was also used to make comparisons. *P*-value < 0.05 was considered statistically significant difference.

## Results

### XYS Improved Body Weight and Food Intake of CUMS-Exposed Rats

**Figure 2:**
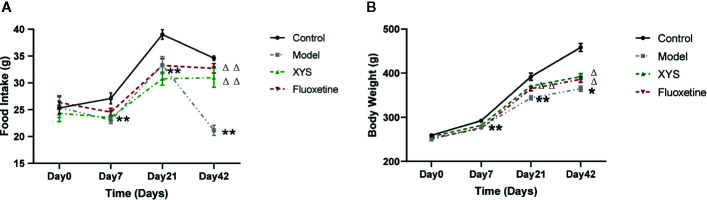
42 days of food intake and body weight were recorded once a week during the modeling period. **(A)** Changes in food intake. **(B)** Changes in body weight. Data were expressed as means ± SEM (n = 20), *P < 0.05, **P < 0.01 versus the control group; ^Δ^ *P* < 0.05, ^ΔΔ^ *P* < 0.01 versus the model group.

**Figure 2A** shows that: on the 7th day of modeling, the food intake of the model group was significantly lower than that of the control group (*P* < 0.01). On the 21st day of CUMS, the rats in the model group were significantly lower than the control group (*P* < 0.01), and there was no significant difference between XYS and fluoxetine group; on the 42nd day of modeling, the rats in the model group were significantly lower than the control group, XYS group and fluoxetine group (*P* < 0.01).

**Figure 2B** shows that: On day 0, there were no significant differences in the bodyweight of the four groups. On the 7th day of CUMS, the body weight of the model group began to be considerably lower than that of the control group (*P* < 0.01). On the 21st day of CUMS, the body weight of the model group was noticeably lower than that of the control group (*P* < 0.01). Compared with the model group, the body weight of the XYS group and the fluoxetine group were statistically different (*P* < 0.01). On the 42nd day of modeling, the body weight of the model group was considerably lower than that of the control group, and there was also a significant difference between the XYS group, the fluoxetine group and the model group (*P* < 0.05).

### XYS Improved Depression-Like Behaviors of CUMS-Treated Rats

**Figure 3:**
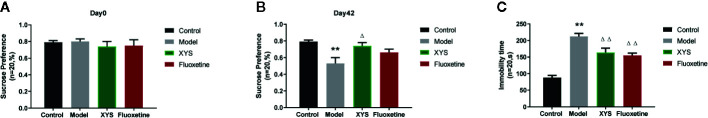
**(A)** SPT of rats in each group at day 0 (baseline); **(B)** SPT of rats in each group at day 42; **(C)** FST of rats in each group at day 42. Data were expressed as means ± SEM (n = 20), **P < 0.01 versus the control group; ^Δ^ *P* < 0.05, ^ΔΔ^ *P* < 0.01 versus the model group.

Continue to observe the behavior of depressed rats, some standard behavioral tests were carried out, including SPT, FST, OFT. From the results of SPT **Figures 3A, B**, it can be seen that the sucrose water consumption of the model group was significantly lower than that of the control group, and there was a significant statistical difference between the two groups (*P* < 0.01). The consumption of sucrose water in the XYS group was significantly higher than that in the model group, and the two were statistically different (*P* < 0.05). The FST is a standard behavioral despair test to evaluate the rat’s depressive-like behaviors, where the immobility time of rats in the CUMS group was significantly longer versus the control group rats at day 42 (*P* < 0.01, **Figure 3C**). It also can be seen that the rats treated with XYS and fluoxetine were significantly lower than those of CUMS group rats (both *P* < 0.01, n = 20). The results show that both XYS and fluoxetine had antidepressant effects.

**Figure 4:**
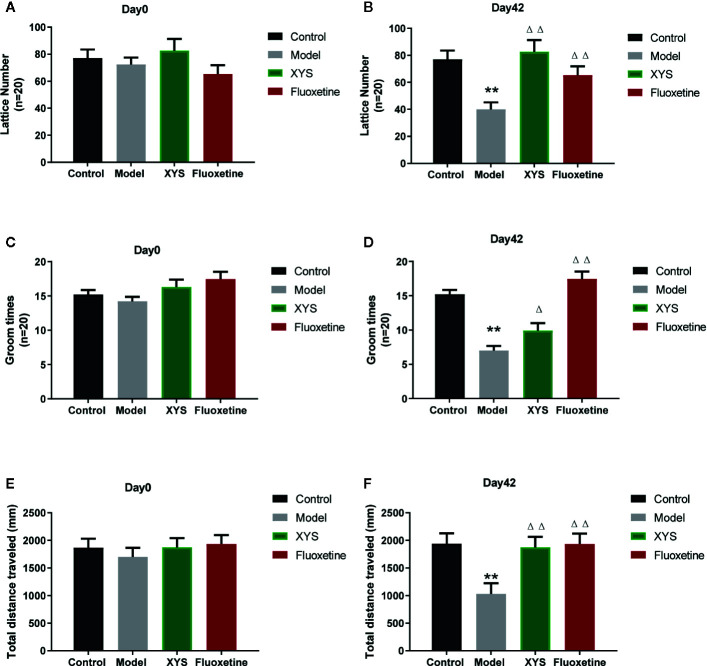
**(A)** The results of lattice number in each group at day 0 (baseline); **(B)** The effects of lattice number in each group at day 42; **(C)** The results of groom times in each group at day 0 (baseline); **(D)** The effects of groom times in each group at day 42; **(E)** The results of total distance in each group at day 0 (baseline); **(F)** The results of total distance in each group at day 42. Data were expressed as means ± SEM (n = 20), **P < 0.01 versus the control group; ^Δ^ *P* < 0.05, ^ΔΔ^ *P* < 0.01 versus the model group.

The OFT can reflect the independent and exploratory behaviors of rats intuitively. It includes the lattice number and groom time. At the beginning of the experiment, there were no significant differences in the results of the OFT at day 0 (both *P* > 0.05, **Figures 4A, C, E**). After CUMS modeling for 42 days, the lattice number, the groom times and the total distance travel of rats in the CUMS group were significantly less than those of control group rats (both *P* < 0.01, **Figures 4B, D, F**), while the XYS and fluoxetine treatment groups were significantly higher than that in the model group, and the two were statistically different (*P* < 0.05).

### XYS Improved the Expression of Serum CORT and Hippocampus Glutamate of CUMS-Exposed Rats

**Figure 5:**
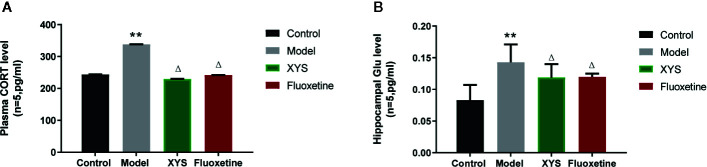
Effects of Xiaoyaosan (XYS) on plasma corticosterone (CORT), hippocampus Glu levels in stress-induced rats. **(A)** Plasma CORT levels; **(B)** Hippocampus Glu levels. Data were expressed as means ± SEM (n = 5), **P < 0.01 versus the control group; ^Δ^ *P* < 0.05 versus the model group.

**Figure 5A** shows that: Versus the control group, the serum CORT content in the model group was significantly higher (*P* < 0.01), and the serum CORT content in the XYS group and the fluoxetine group was considerably lower than that in the model group (*P* < 0.05, *P* < 0.01). **Figure 5B** shows that: Compared with the control group, the Glu content in the hippocampus of the model group was significantly higher (*P* < 0.01), and the content of Glu in the hippocampus of the XYS and fluoxetine groups was significantly lower than that of the model group (*P* < 0.05, *P* < 0.01).

### XYS Improved the Expression of NR2B and GFAP in the Hippocampus of CUMS-Exposed Rats

**Figure 6:**
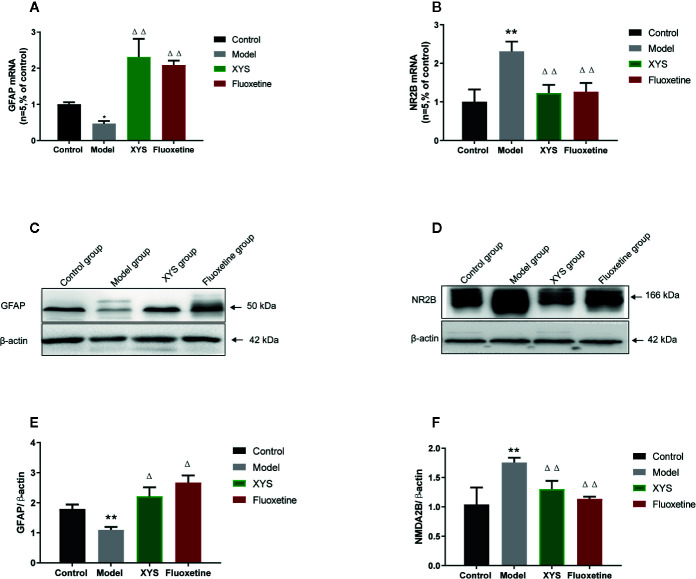
Changes in the hippocampal of glial fibrillary acidic protein (GFAP) and NR2B in chronic unpredictable mild stress (CUMS) rats. **(A)** The mRNA results of GFAP (n = 5); **(B)** The mRNA results of NR2B (n = 5); **(C)** The protein results of GFAP (n = 5); **(D)** The protein results of NMDA2B (n = 5). **(E)** Relative GFAP/β-actin protein; **(F)** Relative NMDA2B/β-actin protein. Data were expressed as means ± SEM (n = 5), **P < 0.01 versus the control group; ^Δ^ *P* < 0.05, ^ΔΔ^ *P* < 0.01 versus the model group.

To continue to detect changes at the molecular level. Detect whether XYS regulated the astrocyte (GFAP) and NMDA receptor system in the CUMS rat model, expressions of GFAP and NR2B were measured. First of all, the RT-qPCR and WB results revealed that the 42-day CUMS modeling could significant reduce the GFAP level in the hippocampus of CUMS rats (both *P* < 0.01, **Figures 6A, B**), and the rats in the two treatment groups showed a significant increase in GFAP level versus the CUMS group rats (*P* < 0.05). The 42-day CUMS modeling could also increase the NR2B level in the hippocampus of CUMS rats (both *P* < 0.01, **Figures 6C–F**), and the rats in the two treatment groups showed a significant decrease in NR2B level versus the CUMS group rats (*P* < 0.05, *P* < 0.01, respectively).

## Discussion

XYS has the effect of soothing the liver and relieving stagnation, nourishing blood, and strengthening the spleen (26). In the treatment of complex diseases caused by chronic stress such as depression, it shows superiority owing to the penetration of traditional Chinese medicine concepts such as overall regulation, syndrome differentiation, and individualized diagnosis and treatment (27). From the macroscopic point of view, the general condition of XYS group rats was significantly better than that of the model group rats. The food intake of XYS group rats was not significantly different from that of the model group on the 7th and 21st day of modeling, but it was considerably higher than that of the model group on the 42nd day (*P* < 0.01). The effect of XYS was also evident from the change in the body weight of rats. From the 7th day, the body weight of XYS group rats was significantly different from that in the model group. After the 21st day of modeling, the rats of the XYS group increased in body weight versus the model group (*P* < 0.05). From the results of body weight and food intake, it can be observed that XYS is a long-term process for regulating body weight and food intake. The effects of sugar water preference test and OFT also indicated that XYS prescript was restraint stress chronically. Fluoxetine was used as a positive control drug, it is widely used first-line antidepressant that selectively inhibits 5-HT transporters and blocks presynaptic membrane uptake (28). The results showed that the food intake, body weight, and sugar water preference of the fluoxetine group were better than the model group (*P* < 0.05).

The functional activities of the central nervous system are based on neurotransmitters and other information molecules (29). In the case of chronic stress, abnormal expression of molecules will cause damage to the brain (30). HPA axis activation is the most critical adaptive and protective response to stress in the body (31). However, in the chronic stress process, sustained HPA axis activation, and high secretion of glucocorticoid (GC) will produce many pathologies of the body (32). In this experiment, we detected a significant increase in the serum CORT content in the model group by Elisa, indicating that the HPA axis is continuously activated. The XYS group and the fluoxetine group reduced the serum CORT content. The HPA axis continues to activate damage to the hippocampus formation mainly through the following pathways: affecting glucose uptake, reducing the expression of glucose carriers, thereby interfering with cell metabolism, reducing cell viability; causing excessive release of excitatory amino acids and increasing extracellular accumulation of glutamate (33); increasing excitatory amino acid N-methyl-D-aspartate (NMDA) receptor-binding protein NR2A, NR2B subunit level, and receptor channel binding site number, thereby increasing hippocampus excitability (34). A large number of studies have shown that when the glutamate content at the synapse is too high. It can cause glutamate to exude synapses and bind to the extra-synaptic NMDA receptor (35). Recent studies have found that NMDA receptors located at the synapse can protect cells, and NMDA receptors outside the synapse stimulate cell cytotoxicity, promote cell necrosis and apoptosis, and NR2B subunits are mainly expressed in sites other than synapses such as cytoplasm and axonal membrane (36, 37). As a result, NR2B is also considered to be a necrosis factor of neurons. By RT-qPCR and WB detection, we can find that both XYS and fluoxetine can down-regulate NR2B at the level of genes and proteins. Thereby protecting neuronal damage.

**Figure 7:**
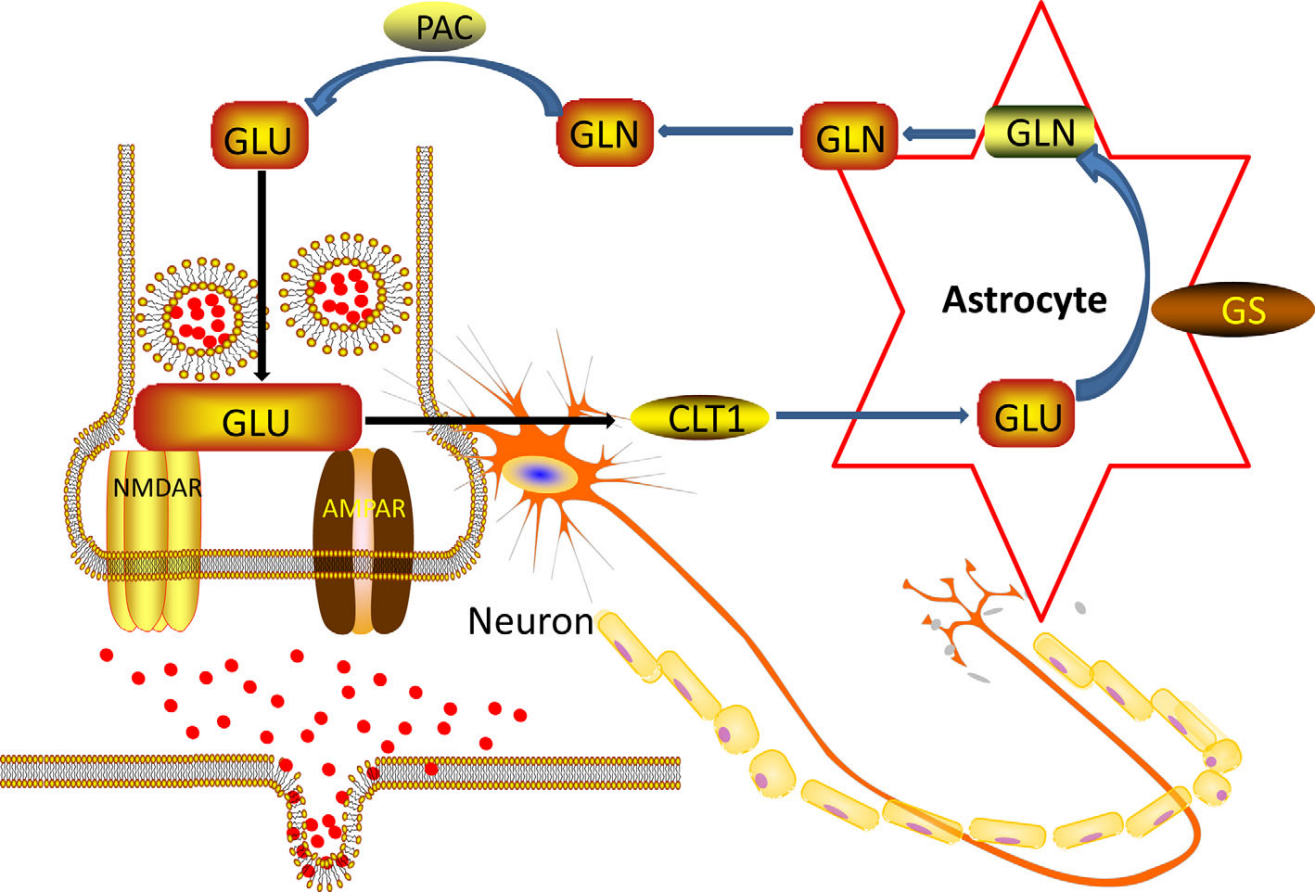
Nerve cell and Ast Glu-Gln loop pattern.

In the nervous system of the brain, astrocyte is about ten times more quantitative than neurons (38). Astrocyte is located between neurons and capillaries. They are an essential part of the blood-brain barrier (39). Astrocyte regulates the transmission of neurotransmitters, regulates glucose metabolism, participates in glutamate metabolism and synaptic plasticity, participates in the regulation of immune mechanisms, and provides neurotrophic support (40). Under normal conditions, the Glu concentration of neuronal cytoplasm is 10 mM/L, the Glu concentration of AC cytoplasm is 50 μM/L to several hundred μM/L, the synaptic gap is 1 μM/L, and the synaptic terminal vesicles can reach 100 mM/L. The concentration of Glu inside and outside the cell varies greatly (41). During synaptic transmission, nerve impulses are transmitted to the synapses, and the nerve endings are depolarized. Synaptic vesicles are released from neurons by synaptic vesicles and plasma membrane fusion (42). Glu receptors acting on the postsynaptic membrane transmit nerve impulses and exert physiological functions. At the same time, they trigger a negative feedback regulation mechanism and are taken up by the glutamate transporter on the astrocyte cell membrane. The glial cells are very strong (43). The ingestion capacity of Glu and glutamine synthetase can convert Glu into glutamine, transport it to the cytoplasm of presynaptic nerve endings, and deamination to Glu by glutaminase. Next, Glu enters the vesicle lumen through the glutamate transporter located on the vehicle and is stored in the vesicle. In resting neurons, Glu is stored in synaptic vesicles at the nerve endings in the form of small membrane-bound organelles (44). Thereby a “glutamate-glutamine cycle” between neurons and glial cells is formed, as showed in **Figure 7** below.

GFAP is mainly expressed in mature astrocyte, and it acts as a backbone protein to support and protect cells. GFAP is a commonly used marker protein of astrocyte and is recorded as a characteristic marker of astrocyte (45). In recent years, GFAP has been involved in many of the biological functions of astrocyte, including maintaining normal physiological functions of the blood-brain barrier, cell proliferation and division, autophagy, vesicles and lysosomes in astrocyte, maintenance of neurotransmitters in astrocytes and neuronal and glial cell balance (38). Besides, there is evidence that only by inhibiting the number of GFAP-positive glial cells is sufficient to induce depressive phase behavior in rats, classic anti-depression drugs such as fluoxetine can act as antidepressants to improve this pathological change and depressive-like behaviour (46).

**Figure 8:**
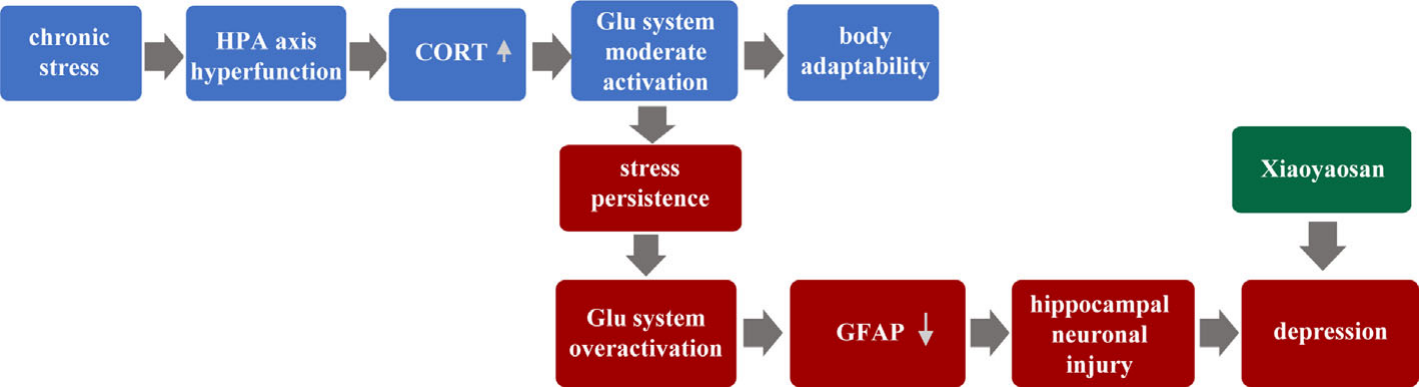
Hypothetical pathogenesis of depression.

In our past experiments, we have found that GFAP in the hippocampus of CUMS mice is down-regulated in the model group, and then XYS and fluoxetine can significantly up-regulate the expression of GFAP in the hippocampus (47). Other animal experiments have also found that different patterns of stimulation in the depression model, the expression in GFAP, and the number of positive cells of GFAP were found to be down-regulated in key brain regions such as the cortex-marginal system and hippocampus (48). We also found the same conclusion. Compared with the control group, the GFAP of the model group showed significant down-regulation of the gene and protein levels, while the XYS group and the fluoxetine group up-regulated the expression of GFAP. Astrocyte is considered to be an essential target in the treatment of depression. A large number of animal studies have shown that different types of antidepressants have different effects on astrocyte. Fluoxetine, a selective serotonin reuptake inhibitor, is effective in inhibiting the reduction in the amount of astrocyte caused by various stimulation. XYS also played a role similar to fluoxetine. So we speculate the pathogenesis of depression, which put forward at the beginning of the article. As shown in **Figure 8** below:

These studies are merely preliminary explorations of the above hypotheses, we preliminary explored the HPA axis, Glu system and astrocyte respectively in this study, but the correlation between the three aspects and the mechanism of causal relationship is not clear. We will continue to conduct further research around these three crucial components of depression. In recent years, the combinations of gas chromatography-quadrupole time of flight mass spectrometry (GC-Q-TOF/MS) and liquid chromatography-quadrupole time of flight mass spectrometry (LC-Q-TOF/MS) has been applied successfully in numerous metabolomics studies to achieve more sensitive and accurate metabolic profiling and screening of biomarkers. The use of GC-Q-TOF/MS is a possible area of future work. Functional electrophysiological research under disease models will help us further reveal the pathogenesis of the disease, we should use them to identify better the metabolite changes and pathways involved and support the proposed hypotheses (49).

## Conclusions

In summary, XYS showed effects in improving the depression-behavior of CUMS rats. The study has proved that XYS could reverse the CUMS-induced changes of the CORT and affect the astrocytic activities and down-regulate the NR2B level in the hippocampus.

## Data Availability Statement

The raw data supporting the conclusions of this article will be made available by the authors, without undue reservation.

## Ethics Statement

The animal study was reviewed and approved by the Institutional Animal Care and Use Committee at Beijing University of Chinese Medicine.

## Author Contributions

Conceived and designed the experiments: MS, JC and JZ. Carried out the animal experiment: MS, XL, TW, and YL. Conducted molecular experiment: MS, TW and YZ. Analyzed the data: MS. All authors contributed to the article and approved the submitted version.

## Funding

This research was supported by grants from the National Natural Science Foundation of China (No. 81973748, 81630104), and Huang Zhendong Research Fund for Traditional Chinese Medicine of Jinan University (No. 201911).

## Conflict of Interest

The authors declare that the research was conducted in the absence of any commercial or financial relationships that could be construed as a potential conflict of interest.
